# Prioritization of Kidney Cell Types Highlights Myofibroblast Cells in Regulating Human Blood Pressure

**DOI:** 10.1016/j.ekir.2024.03.001

**Published:** 2024-03-13

**Authors:** Mahboube Ganji-Arjenaki, Zoha Kamali, International Consortium of Blood Pressure, Evangelos Evangelou, Helen R. Warren, He Gao, Georgios Ntritsos, Niki Dimou, Tonu Esko, Reedik Mägi, Lili Milani, Peter Almgren, Thibaud Boutin, Stéphanie Debette, Jun Ding, Franco Giulianini, Elizabeth G. Holliday, Anne U. Jackson, Ruifang Li -Gao, Wei -Yu Lin, Jian'an Luan, Massimo Mangino, Christopher Oldmeadow, Bram Peter Prins, Yong Qian, Muralidharan Sargurupremraj, Nabi Shah, Praveen Surendran, Sébastien Thériault, Niek Verweij, Sara M. Willems, Jing -Hua Zhao, Philippe Amouyel, John Connell, Renée de Mutsert, Alex S.F. Doney, Martin Farrall, Cristina Menni, Andrew D. Morris, Raymond Noordam, Guillaume Paré, Neil R. Poulter, Denis C. Shields, Alice Stanton, Simon Thom, Gonçalo Abecasis, Najaf Amin, Dan E. Arking, Kristin L. Ayers, Caterina M. Barbieri, Chiara Batini, Joshua C. Bis, Tineka Blake, Murielle Bochud, Michael Boehnke, Eric Boerwinkle, Dorret I. Boomsma, Erwin P. Bottinger, Peter S. Braund, Marco Brumat, Archie Campbell, Harry Campbell, Aravinda Chakravarti, John C. Chambers, Ganesh Chauhan, Marina Ciullo, Massimiliano Cocca, Francis Collins, Heather J. Cordell, Gail Davies, Martin H. de Borst, Eco J. de Geus, Ian J. Deary, Joris Deelen, Fabiola Del Greco M, Cumhur Yusuf Demirkale, Marcus Dörr, Georg B. Ehret, Roberto Elosua, Stefan Enroth, A. Mesut Erzurumluoglu, Teresa Ferreira, Mattias Frånberg, Oscar H. Franco, Ilaria Gandin, Paolo Gasparini, Vilmantas Giedraitis, Christian Gieger, Giorgia Girotto, Anuj Goel, Alan J. Gow, Vilmundur Gudnason, Xiuqing Guo, Ulf Gyllensten, Anders Hamsten, Tamara B. Harris, Sarah E. Harris, Catharina A. Hartman, Aki S. Havulinna, Andrew A. Hicks, Edith Hofer, Albert Hofman, Jouke-Jan Hottenga, Jennifer E. Huffman, Shih-Jen Hwang, Erik Ingelsson, Alan James, Rick Jansen, Marjo -Riitta Jarvelin, Roby Joehanes, Åsa Johansson, Andrew D. Johnson, Peter K. Joshi, Pekka Jousilahti, J. Wouter Jukema, Antti Jula, Mika Kähönen, Sekar Kathiresan, Bernard D. Keavney, Kay-Tee Khaw, Paul Knekt, Joanne Knight, Ivana Kolcic, Jaspal S. Kooner, Seppo Koskinen, Kati Kristiansson, Zoltan Kutalik, Maris Laan, Marty Larson, Lenore J. Launer, Benjamin Lehne, Terho Lehtimäki, David C.M. Liewald, Li Lin, Lars Lind, Cecilia M. Lindgren, YongMei Liu, Ruth J.F. Loos, Lorna M. Lopez, Yingchang Lu, Leo-Pekka Lyytikäinen, Anubha Mahajan, Chrysovalanto Mamasoula, Jaume Marrugat, Jonathan Marten, Yuri Milaneschi, Anna Morgan, Andrew P. Morris, Alanna C. Morrison, Peter J. Munson, Mike A. Nalls, Priyanka Nandakumar, Christopher P. Nelson, Teemu Niiranen, Ilja M. Nolte, Teresa Nutile, Albertine J. Oldehinkel, Ben A. Oostra, Paul F. O'Reilly, Elin Org, Sandosh Padmanabhan, Walter Palmas, Aarno Palotie, Alison Pattie, Brenda W.J.H. Penninx, Markus Perola, Annette Peters, Ozren Polasek, Peter P. Pramstaller, Quang Tri Nguyen, Olli T. Raitakari, Rainer Rettig, Kenneth Rice, Paul M. Ridker, Janina S. Ried, Harriëtte Riese, Samuli Ripatti, Antonietta Robino, Lynda M. Rose, Jerome I. Rotter, Igor Rudan, Daniela Ruggiero, Yasaman Saba, Cinzia F. Sala, Veikko Salomaa, Nilesh J. Samani, Antti-Pekka Sarin, Reinhold Schmidt, Helena Schmidt, Nick Shrine, David Siscovick, Albert V. Smith, Harold Snieder, Siim Sõber, Rossella Sorice, John M. Starr, David J. Stott, David P. Strachan, Rona J. Strawbridge, Johan Sundström, Morris A. Swertz, Kent D. Taylor, Alexander Teumer, Martin D. Tobin, Maciej Tomaszewski, Daniela Toniolo, Michela Traglia, Stella Trompet, Jaakko Tuomilehto, Christophe Tzourio, André G. Uitterlinden, Ahmad Vaez, Peter J. van der Most, Cornelia M. van Duijn, Germaine C. Verwoert, Veronique Vitart, Uwe Völker, Peter Vollenweider, Dragana Vuckovic, Hugh Watkins, Sarah H. Wild, Gonneke Willemsen, James F. Wilson, Alan F. Wright, Jie Yao, Tatijana Zemunik, Weihua Zhang, John R. Attia, Adam S. Butterworth, Daniel I. Chasman, David Conen, Francesco Cucca, John Danesh, Caroline Hayward, Joanna M.M. Howson, Markku Laakso, Edward G. Lakatta, Claudia Langenberg, Olle Melander, Dennis O. Mook-Kanamori, Colin N.A. Palmer, Lorenz Risch, Robert A. Scott, Rodney J. Scott, Peter Sever, Tim D. Spector, Pim van der Harst, Nicholas J. Wareham, Eleftheria Zeggini, Daniel Levy, Patricia B. Munroe, Christopher Newton-Cheh, Morris J. Brown, Andres Metspalu, Bruce M. Psaty, Louise V. Wain, Paul Elliott, Mark J. Caulfield, Soroush Sardari, Martin de Borst, Harold Snieder, Ahmad Vaez

**Affiliations:** 1 Drug Design and Bioinformatics Unit, Department of Medical Biotechnology, Biotechnology Research Center, Pasteur Institute of Iran, Tehran, Iran; 2 Department of Molecular Medicine, School of Advanced Technologies, Shahrekord University of Medical Sciences, Shahrekord, Iran; 3 Department of Epidemiology, University of Groningen, University Medical Centre Groningen, Groningen, The Netherlands; 4 Department of Bioinformatics, Isfahan University of Medical Sciences, Isfahan, Iran; 5 Division of Nephrology, Department of Internal Medicine, University of Groningen, University Medical Center Groningen, Groningen, The Netherlands

**Keywords:** blood pressure, cell-type, genome, kidney myofibroblast, scRNA-seq

## Abstract

**Introduction:**

Blood pressure (BP) is a highly heritable trait with over 2000 underlying genomic loci identified to date. Although the kidney plays a key role, little is known about specific cell types involved in the genetic regulation of BP.

**Methods:**

Here, we applied stratified linkage disequilibrium score (LDSC) regression to connect BP genome-wide association studies (GWAS) results to specific cell types of the mature human kidney. We used the largest single-stage BP genome-wide analysis to date, including up to 1,028,980 adults of European ancestry, and single-cell transcriptomic data from 14 mature human kidneys, with mean age of 41 years.

**Results:**

Our analyses prioritized myofibroblasts and endothelial cells, among the total of 33 annotated cell type, as specifically involved in BP regulation (*P* < 0.05/33, i.e., 0.001515). Enrichment of heritability for systolic BP (SBP) was observed in myofibroblast cells in mature human kidney cortex, and enrichment of heritability for diastolic BP (DBP) was observed in descending vasa recta and peritubular capillary endothelial cells as well as stromal myofibroblast cells. The new finding of myofibroblast, the significant cell type for both BP traits, was consistent in 8 replication efforts using 7 sets of independent data, including in human fetal kidney, in East-Asian (EAS) ancestry, using mouse single-cell RNA sequencing (scRNA-seq) data, and when using another prioritization method.

**Conclusion:**

Our findings provide a solid basis for follow-up studies to further identify genes and mechanisms in myofibroblast cells that underlie the regulation of BP.

## Graphical abstract

**Figure:**
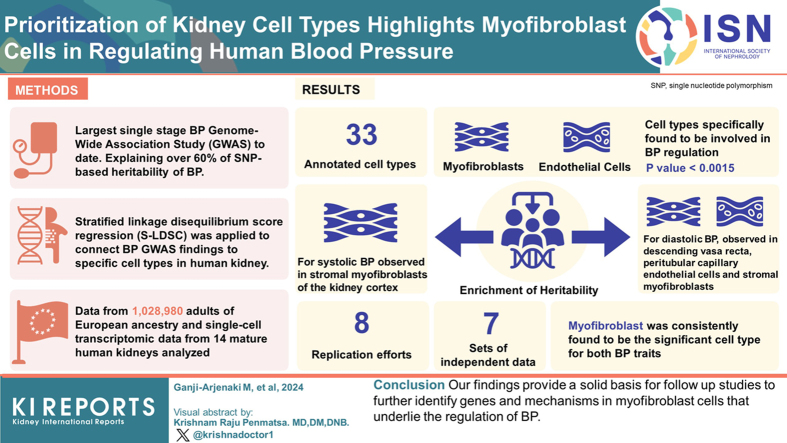

BP is a highly complex, polygenic, and heritable trait for which over 2000 genomic loci have been identified in our most recent meta-analysis of GWAS. These associations explain about 60% of common single nucleotide variation (SNV, formerly SNP) heritability of SBP and DBP.^1^ Biological interpretation of GWAS results remains challenging. The majority of the identified variants are located in noncoding regions and linkage disequilibrium present in the human genome confounds efforts to pinpoint causal variants and genes. We and others have recently applied post-GWAS analyses of BP traits aiming to translate the identified genomic associations to biological insights. These results highlight key regulatory genes and mechanisms in cardiovascular tissues,^1^ blood^2^ and the kidney.^3^ However, a clear picture of cell types involved in regulating BP phenotypes is still elusive.

The kidney, as a key player in regulating BP, is a highly complex organ and plays a critical role in homeostasis. This includes controlling blood electrolytes and acid-base balance and also secreting hormones that regulate blood composition and BP.^4^ Anatomically, each kidney is composed of cortex, medulla, and pelvic parts. Stewart and colleagues, using scRNA-seq, identified that these anatomic parts in fetal and mature kidneys include 4 clusters—endothelial, immune, fibroblast and myofibroblast, and epithelium cells—by loading highly variable genes into a principal component analysis.^5^

In recent years, scRNA-seq technologies have enabled sequencing thousands of cells within specific organs with high quality. Compared to the traditional bulk RNA sequencing, scRNA-seq can characterize transcriptome profiles of specific cell types with much higher resolution.^6^ These cell-type–specific transcriptome profiles can then be used to determine which genes show cell-type–specific expression patterns. Integration of such data with GWAS results may help to discover relevance of specific cell types for human complex traits.^7^

A previously published study of scRNA-seq data of mouse kidneys reported about 25 cell-type annotations from glomerular and tubular compartments.^8^ On the basis of this cell-type reference, deconvolution of human kidney RNA sequencing data and integration with a GWAS of SBP in 477,054 individuals highlighted endothelial cells of the distal tubule in SBP regulation.^9^ Recently, Stewart and colleagues have generated 2 separate comprehensive scRNA-seq data sets of mature and fetal human kidneys, consisting of immune, vasculature, nephron, and stroma functional compartments.^5^ This study has analyzed data from 14 human kidneys and reports over 33 cell-type annotations in mature kidneys.^5^ The availability of such a data set as well as the newer larger GWAS study^1^ warrant more efforts to elucidate key cell types in BP phenotypes.

Here, we integrate the most recent, largest GWAS of BP traits with a comprehensive kidney scRNA-seq data set to understand which kidney cell types are key in BP regulation. We then tried to replicate our discovery findings in several replication sets by using either different independent data sets or applying an alternative computational method (Figure 1).

**Figure 1:**
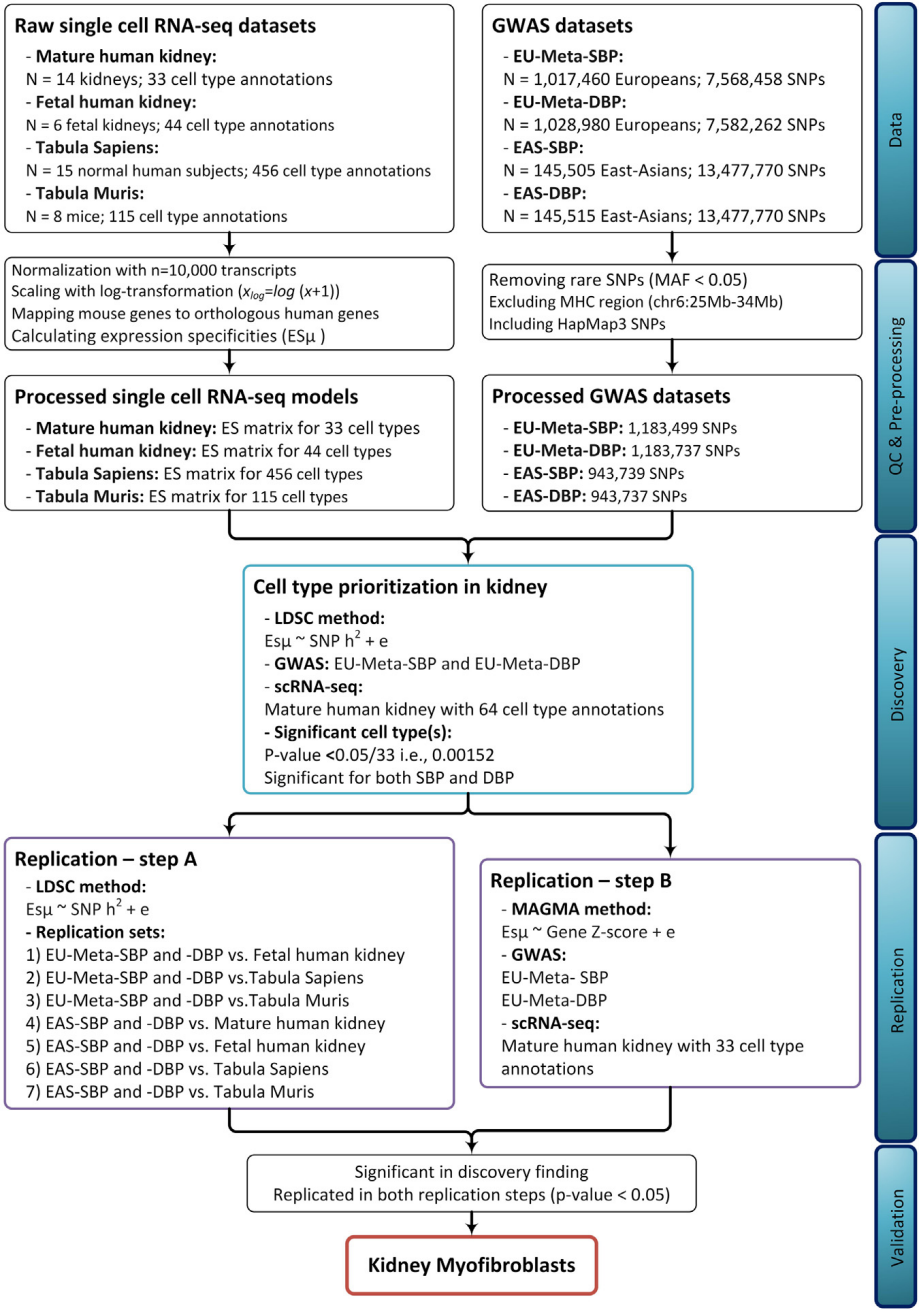
Flow diagram depicting the design of this study. Replication Step A entails replicating the discovery findings using 7 independent combinations of GWAS and single-cell RNA sequencing datasets, including mature and fetal human kidney datasets, as well as Tabula Sapiens and Muris datasets. These datasets are described in the references. Replication Step B involves replicating the discovery findings using another computational method, specifically MAGMA. EU-Meta-SBP and -DBP GWASs are the meta-analysis results of over 1 million individuals from European ancestry. EAS-SBP and EAS-DBP are GWAS results of blood pressure traits in over 145,000 East-Asian individuals. DBP, diastolic blood pressure; EAS, East-Asian; EU, European; GWAS, genome-wide association studies; MAGMA, Multi-marker Analysis of GenoMic Annotation; SBP, systolic blood pressure.

## Methods

We prioritized human kidney cell types playing a role in BP in a single discovery set and then tried to confirm the results in 7 independent replication sets. For further confirmation, we tried to replicate the discovery results using an alternative computational method (Figure 1).

### GWAS Data

For discovery, we used the results of the latest, largest GWAS meta-analysis of SBP and DBP to date, including up to 1,028,980 adults of European ancestry.^1^ Then, for independent replication, we used GWAS results of the same traits from up to 145,000 individuals of EAS ancestry.^10^

### scRNA-Seq Data Sets

We used scRNA-seq data from 4 different data sets. For discovery, we used data from Stewart *et al.* on 33 cell-type annotations from the cortex and medulla of 14 mature human kidneys. We used expression matrix of full kidney, for which we performed preprocessing and cell-type prioritization steps. For replication in independent scRNA-seq data sets, we used the following: (i) fetal human kidney data from Stewart *et al.*;^5^ this data set includes 44 cell-type annotations derived from 6 fetal kidneys at 7 to 16 weeks postconception; (ii) Tabula Sapiens, a human cell atlas from 24 organs of 15 normal human subjects resulting in 456 cell-type annotations;^11^ and (iii) Tabula Muris data, a compendium of single-cell transcriptome data from the model organism *Mus musculus*, containing 115 cell types from 20 organs.^12^

### GWAS Data Preprocessing

We adapted all GWAS summary statistics using the script “munge_sumstats.py” from the LDSC v1.0.0 package for cell-type prioritization.^13^ All prepared GWAS data sets were restricted to HapMap3 SNVs, excluding SNVs in the major histocompatibility complex region (chr6:25Mb–34Mb).

### scRNA-Seq Data Preprocessing

All scRNA-seq data sets were annotated to cell types using the original publications, which are based on cell-type–specific markers^5^ and were then processed uniformly following the pipeline implemented in the CELL type expression-specificity tool kit.^14^ First, expression values from a raw gene expression matrix were normalized to a common transcript count, with *n* = 10,000 transcripts as a scaling factor, and log-transformation (x_log_= log [x+1]) was applied. Next, we performed mouse-human ortholog mapping using Ensembl (v. 91)^14^ and retained only the genes with 1–1 orthologs.

Expression specificity (ES) matrices were calculated separately for the mature and fetal human kidney, Tabula Sapiens, and Tabula Muris data sets. Cell-type ES weights (ES_w_) were calculated using the following 4 ES metrics: (i) gene enrichment score,^15^ (ii) expression proportion,^16^ (iii) normalized specificity index,^17^ and (iv) differential expression *t*-statistic.^14^ For each ES metric, we separately computed gene-specific ES_w_ values for all genes. Then, average ES scores (ES_μ_; range [0–1]; low to high cell-type specificity) based on the 4 ES metrics were calculated. All cell-type prioritization results were computed based on the ES_μ_ estimates.^14^

### Cell-Type Prioritization

We performed cell-type prioritization analyses based on the pipeline implemented in CELLECT (CELL type Expression-specific integration for Complex Traits) tool kit^14^ using 2 different algorithms described next.

#### Discovery Step – Based on LDSC Regression Analysis

Partitioned LDSC was used to test whether specific genes of each cell type, based on the average specificity metrics described earlier, were enriched in heritability for the BP traits. To capture most regulatory elements that could contribute to the effect of the region on the trait, a 100 kb window surrounding the genes’ transcribed region was used. The maximum ES_μ_ value was assigned when a variant within the 100 kb window overlapped with multiple genes. An annotation file for each cell type was constructed, by assigning gene ES_μ_ values to variants within a 100 kb window of the genes. Ensembl v. 91 (hg19) was used as the reference genome for genetic variant and gene chromosomal positions and 1000 Genomes Project SNVs as baseline model used in stratified LDSC by default.^14^ LDSCs were computed for HapMap3 SNVs for each annotation. Stratified LDSC was run with default settings, then 1-tailed *P*-values for positive associations between the trait heritability and cell-type ES_μ_ values were reported. Regression effect size was estimated for each cell type which represents the change in per-SNV heritability due to increase in ES (ES_μ_) for the given cell-type annotation and standard errors of effect sizes were reported using the jackknife method.^14^ We used Bonferroni correction to control multiple testing of the multiple cell types in the discovery data set.

#### Assessing Causal Effects of Significant Cell Type on BP

To assess the causal effect of significant cell type on BP, we conducted a 2-sample Mendelian randomization analysis.^18^ This analytic approach is developed to assess causality from exposure to outcome, using genetic associations contributing to the outcome through the exposure. Kidney expression level of a cell-type–specific marker gene (*ACTA2*) was considered as the exposure. Therefore, summary statistics of SNVs with regulatory effects on its gene expression level in kidney (eQTLs; expression Quantitative Trait Loci)^19^ was used as the exposure instrumental variables. Summary statistics of meta-GWASs of BP traits from Warren *et al.*^1^ were used as the outcome data sets.

#### Replication Step

Cell types that were significantly prioritized for both SBP and DBP were selected for replication using 2 different strategies as follows: first by changing the input data sets and analyzing new independent pairs of GWAS and scRNA-seq data, including GWAS of another ancestry and scRNA-seq data of other organisms; and second, by changing the analysis algorithm. For replications, we considered a nominal significance level of 0.05.

##### Replication A - Using Independent Combinations of GWAS and scRNA-Seq Data

We replicated CELLECT-LDSC regression for selected cell types using 7 independent combinations of GWAS and scRNA-seq data as follows: (i) European meta-GWAS results^1^ with fetal kidney data,^5^ (ii) European meta-GWAS results^1^ with Tabula Sapiens cell atlas data,^11^ (iii) European meta-GWAS results^1^ with Tabula Muris data,^12^ (iv) East-Asian GWAS results^10^ with mature human kidney data,^5^ (v) East-Asian GWAS results^10^ with fetal kidney data set,^5^ (vi) East-Asian GWAS results^10^ with Tabula Sapiens cell atlas data,^11^ and (vii) East-Asian GWAS results^10^ with Tabula Muris data.^12^

##### Replication B - Using MAGMA, as an Alternative Cell Type Association Algorithm

MAGMA is an algorithm for gene-set analysis using GWAS summary statistics^20^ that can be used as an alternative gene-level approach to assess the robustness of the SNV-level stratified LDSC cell-type prioritization.^14^ Briefly, MAGMA computes a gene-level association statistic by averaging *P*-values transformed to a *z*-value of SNVs within 100 kb of the gene at either side while taking into account LD structure based on the European reference panel of the 1000 genomes project phase 3 as the reference population.

Gene-level *z*-statistics were corrected for the following default MAGMA covariates: gene size, gene density, mean sample size for tested SNVs per gene, the inverse of the minor allele counts per gene, and the log of these metrics. Next, a regression model was fitted using MAGMA gene-level *z*-statistics as the dependent variable and cell-type ES_μ_ as the independent variable. Cell-type prioritization *P*-values (from the linear regression model) were reported for the cell-type ES_μ_ regression coefficients to BP gene-level *z*-statistics.^14^

## Results

### Discovery of Kidney Cell-Type Specificity of BP Genetic Associations

We leveraged scRNA-seq data from cortex and medulla of mature human kidney, with a total of 33 cell-type annotations,^5^ to systematically map BP traits to relevant cell types. To this end, we used the largest available GWAS of BP by Warren *et al.* who identified over 2000 genomic loci regulating BP traits in up to 1,028,980 individuals.^1^ We used the LDSC regression statistical method to connect BP GWAS results to specific cell types. LDSC assessed enrichment of the common SNV heritability of BP traits in the cell-type–specific genes of mature human kidneys.^13^

**Figure 2:**
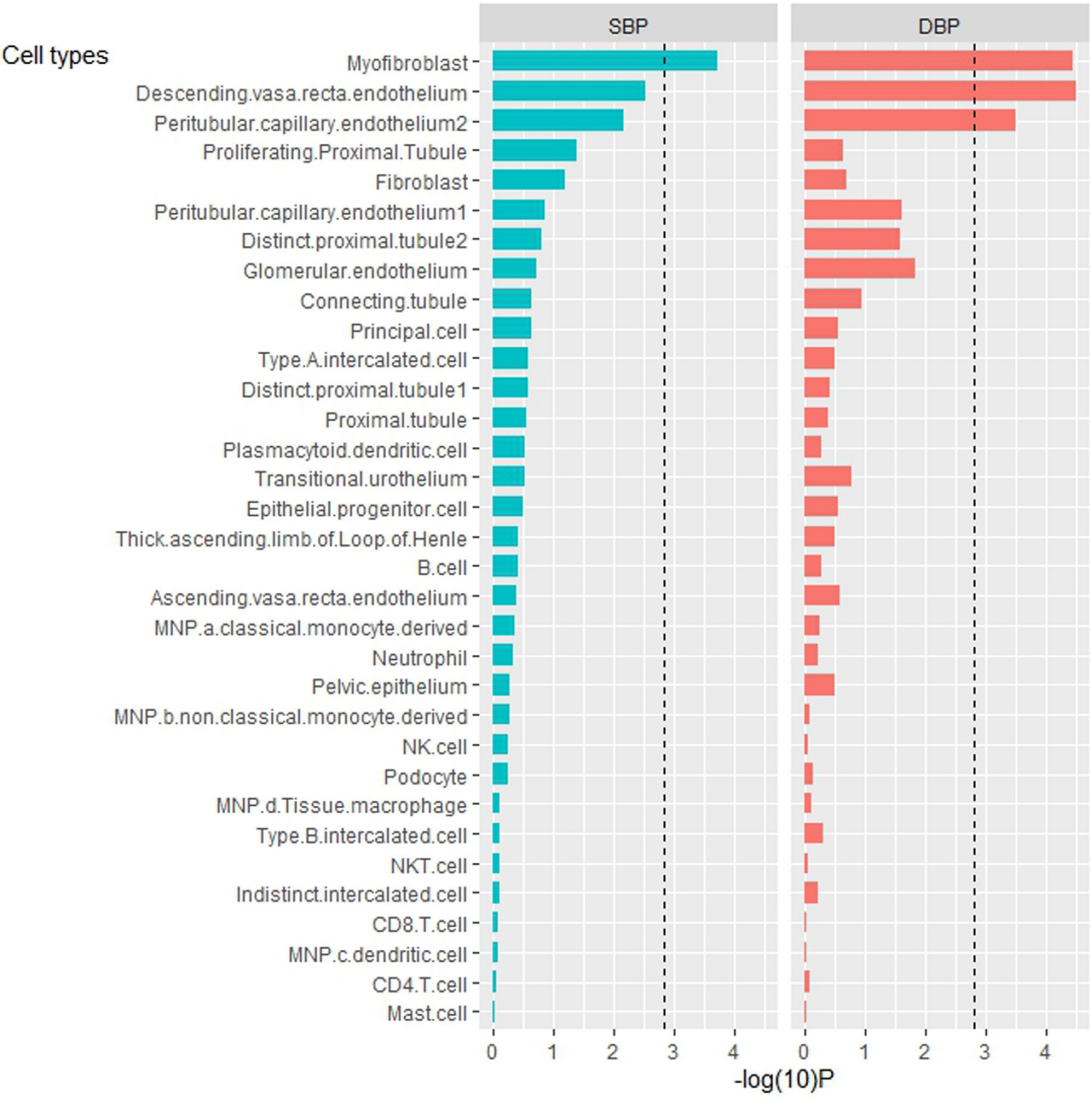
Discovery cell-type prioritization estimated by LDSC in systolic and diastolic BP. Cell-type prioritization results are generated using mature human kidney data. Bars represent the strength of association (−log [10] *P*) of cell-type expression specificity with heritability for BP phenotypes. Cell types are listed based on −log (10) P in descending order for SBP. The dashed black line indicates the Bonferroni corrected significance threshold, that is, *P*-value < 0.05/33. BP, blood pressure; DBP, diastolic blood pressure; LDSC, linkage disequilibrium score; SBP, systolic blood pressure.

Our discovery cell-type prioritization of mature human kidney for SBP and DBP by the LDSC method (Figure 2 and Supplementary Table S1) highlights 2 cell types from cortex as significantly associated with BP traits, namely myofibroblast and endothelial cells (*P* < 1.51 × 10^‒3^, i.e., 0.05/33) (Table 1).

**Table 1:** Significant results of discovery analysis listed as prioritized cell types estimated by LDSC in blood pressure related phenotypes

scRNA-seq data set	Organ	GWAS	Cell type	Beta	SE	*P*-value
Human kidney cell atlas^5^	Mature kidney	EU-Meta-SBP^1^	Myofibroblast	1.17 × 10^−8^	3.30 × 10^−9^	**1.88 × 10**^**−4**^
		EU-Meta-DBP^1^	Descending vasa recta endothelium	1.22 × 10^−8^	3.05 × 10^−9^	**3.12 × 10**^**−5**^
			Myofibroblast	1.29 × 10^−8^	3.26 × 10^−9^	**3.65 × 10**^**−5**^
			Peritubular capillary endothelium2^a^	1.02 × 10^−8^	2.99 × 10^−9^	**3.21 × 10**^**−4**^

DBP, diastolic blood pressure; EU, European; GWAS, genome-wide association studies; LDSC, linkage disequilibrium score; SBP, systolic blood pressure; scRNA-seq, single-cell RNA sequencing.

This table shows the Beta, SE, and *P*-value for the LDSC integration of EU-Meta-SBP and EU-Meta-DBP GWASs with mature human kidney cell types. Bonferroni corrected significant associations (*P* < 1.51 × 10^–^^3^) are shown in bold.

a Distinct subsets of the same cell type, based on canonical marker expression, are indicated by suffixes 2 as in the original publications.

### Assessing Causal Effects of Significant Cell Type on BP

Kidney expression level of the *ACTA2* gene, as a specific marker for myofibroblast cell type, was considered as the exposure. Interestingly, we found that each SD increase in the expression levels of *ACTA2* was causally associated with a 0.2 mm Hg increase in SBP (*P* = 0.004), whereas its association with DBP was nonsignificant.

### Replication Step A - In Other Data Sets

We followed-up with myofibroblasts, as the only significant finding for both SBP and DBP, by further replication efforts. We tried to replicate this finding by applying the same computational method, that is, LDSC, but using independent combinations of GWAS and scRNA-seq data sets ending up with 7 rounds of replication (Table 2). For replication, we considered a nominal significance level of 0.05.

**Table 2:** Replication step A - myofibroblast association with SBP and DBP in other data sets

GWAS	Data set	Organ	Cell type	Beta	SE	*P*-value
EU_Meta-SBP^1^	Human kidney cell atlas/fetal^5^	Fetal kidney	Proliferating myofibroblast	1.26 × 10^−8^	3.75 × 10^−9^	**3.83 × 10**^**−4**^
			Myofibroblast2^a^	1.22 × 10^−8^	3.76 × 10^−9^	**5.56 × 10**^**−4**^
			Myofibroblast1^a^	8.88 × 10^−9^	3.15 × 10^−9^	**2.41 × 10**^**−3**^
EU_Meta-DBP^1^			Myofibroblast2^a^	1.63 × 10^−8^	3.98 × 10^−9^	**2.18 × 10**^**−5**^
			Proliferating myofibroblast	1.46 × 10^−8^	4.02 × 10^−9^	**1.37 × 10**^**−4**^
			Myofibroblast1^a^	1.03 × 10^−8^	3.44 × 10^−9^	**1.36 × 10**^**−3**^
EU_Meta-SBP^1^	Tabula Sapiens^11^	Fat	Myofibroblast	1.32 × 10^−8^	3.97 × 10^−9^	**4.31 × 10**^**−4**^
		Bladder	Myofibroblast	5.71 × 10^−9^	2.57 × 10^−9^	**1.32 × 10**^**−2**^
		Lung	Myofibroblast	5.53 × 10^−9^	4.22 × 10^−9^	9.49 × 10^−2^
EU_Meta-DBP^1^		Fat	Myofibroblast	1.70 × 10^−8^	3.93 × 10^−9^	**7.66 × 10**^**−6**^
		Lung	Myofibroblast	5.01 × 10^−9^	4.01 × 10^−9^	1.06 × 10^−1^
		Bladder	Myofibroblast	2.54 × 10^−9^	2.55 × 10^−9^	1.59 × 10^−1^
EU_Meta-SBP^1^	Tabula Muris^12^	Heart	Myofibroblast	1.67 × 10^−8^	4.06 × 10^−9^	**1.84 × 10**^**−5**^
EU_Meta-DBP^1^			Myofibroblast	1.68 × 10^−8^	4.19 × 10^−9^	**2.93 × 10**^**−5**^
EAS_SBP^10^	Human kidney cell atlas/mature^5^	Mature kidney	Myofibroblast	1.71 × 10^−8^	4.76 × 10^−9^	**1.61 × 10**^**−4**^
EAS_DBP^10^			Myofibroblast	1.38 × 10^−8^	4.24 × 10^−9^	**5.64 × 10**^**−4**^
EAS_SBP^10^	Human kidney cell atlas/fetal^5^	Fetal kidney	Proliferating myofibroblast	1.80 × 10^−8^	5.17 × 10^−9^	**2.56 × 10**^**−4**^
			Myofibroblast2^a^	9.45 × 10^−9^	4.86 × 10^−9^	**2.59 × 10**^**−2**^
			Myofibroblast1^a^	6.49 × 10^−9^	4.18 × 10^−9^	6.04 × 10^−2^
EAS_DBP^10^			Proliferating myofibroblast	1.48 × 10^−8^	4.55 × 10^−9^	**5.80 × 10**^**−4**^
			Myofibroblast1^a^	6.07 × 10^−9^	3.96 × 10^−9^	6.27 × 10^−2^
			Myofibroblast2^a^	5.97 × 10^−9^	4.14 × 10^−9^	7.48 × 10^−2^
EAS_SBP^10^	Tabula Sapiens^11^	Fat	Myofibroblast	1.12 × 10^−8^	4.99 × 10^−9^	**1.27 × 10**^**−2**^
		Lung	Myofibroblast	2.70 × 10^−10^	4.89 × 10^−9^	4.78 × 10^−1^
		Bladder	Myofibroblast	−2.68 × 10^−9^	4.11 × 10^−9^	7.43 × 10^−1^
EAS_DBP^10^		Fat	Myofibroblast	1.22 × 10^−8^	4.90 × 10^−9^	**6.48 × 10**^**−3**^
		Bladder	Myofibroblast	−2.59 × 10^−9^	3.37 × 10^−9^	7.79 × 10^−1^
		Lung	Myofibroblast	−3.99 × 10^−9^	4.01 × 10^−9^	8.40 × 10^−1^
EAS_SBP^10^	Tabula Muris^12^	Heart	Myofibroblast	1.81 × 10^−8^	5.02 × 10^−9^	**1.51 × 10**^**−4**^
EAS_DBP^10^			Myofibroblast	1.32 × 10^−8^	4.69 × 10^−9^	**2.35 × 10**^**−3**^

DBP, diastolic blood pressure; EAS, East-Asian; EU, European; GWAS, genome-wide association studies; LDSC, linkage disequilibrium score; SBP, systolic blood pressure.

This table shows the Beta, SE, and *P*-value for the LDSC integration of GWAS summary statistics of (European ancestry) EU-Meta-BP and (East Asian ancestry) EAS-BP traits with additional human and mouse single-cell RNA sequencing data sets. Nominally significant associations (*P* < 0.05) are shown in bold.

a Distinct subsets of a cell type, based on canonical marker expression, are indicated by suffixes 1 and 2 as in the original publications.

We first used an independent comprehensive fetal human kidney scRNA-seq data set, including 44 cell types from 6 fetal human kidneys obtained at 7 to 16 weeks postconception.^5^ All available myofibroblast cell types were significantly replicated for BP traits.

Second, we evaluated a human single-cell atlas of Tabula Sapiens consortium (from 24 organs of 15 normal human subjects resulting in *n* = 456 cell types)^11^ for BP traits. The associations of myofibroblast cells with SBP and DBP were replicated in myofibroblast of fat organs. In addition, myofibroblast cells in bladder were significantly associated with SBP but not with DBP. No myofibroblast cell type of the lung reached our significance threshold using specificity metrics in this data set. Unfortunately, myofibroblasts from kidney were not available in the list of annotated cell types in this human single-cell atlas.

Third, we evaluated a compendium of single-cell transcriptome data from the model organism *Mus musculus* consisting of 115 different cell types from 20 organs.^12^ We found that SBP and DBP were significantly associated with myofibroblast cell types from the heart. Similar to the above data set, myofibroblasts were not available for the kidney in the list of annotated cell types.

Fourth, we changed our GWAS data set and used SBP and DBP GWAS data from up to 145,000 EAS individuals^10^ and analyzed them with the mature human kidney data set from the discovery step. Similarly, all myofibroblast cell types associated with BP traits were significantly replicated in the EAS population.

Fifth, we evaluated the fetal human kidney scRNA-seq data set with EAS-BP traits GWASs. Two-thirds of myofibroblast cell types from fetal kidney were significantly associated with EAS-SBP.

Sixth, we found a significant association of only fat myofibroblast cell type in Tabula Sapiens data set with EAS-BP traits, similar to our finding from analysis of EU-Meta-BP traits with this data set.

Seventh, again our finding from evaluation of the Tabula Muris data set with EU-Meta-BP GWAS data was replicated, such that the association of heart myofibroblast cells with EAS-BP traits was significant.

In summary, our major discovery finding, myofibroblast cells from the mature human kidney, were repeatedly replicated in independent human and mouse single-cell transcriptome studies as well as GWAS data from another ancestry, that is, EAS (Table 2).

### Replication step B - using an alternative computational method

For further confirmation, we also followed-up with our major discovery finding, namely myofibroblasts, and applied MAGMA as an alternative computational method for replication.^20^ MAGMA evaluated linear relationship of gene-level genetic associations with BP traits and ES for cell types in mature human kidneys. Here, we used the same GWAS and scRNA-seq data sets as in the discovery analyses.^1^^,^^5^ Again, we considered a nominal significance level of 0.05 for replication. MAGMA significantly confirmed the association of myofibroblast cells with both SBP and DBP (Table 3).

**Table 3:** Replication step B - using MAGMA for assessing myofibroblast association with SBP and DBP

Cell type	SBP			DBP		
	Beta	SE	*P*-value	Beta	SE	*P*-value
Myofibroblast	8.03 × 10^−1^	7.49 × 10^−2^	**4.80 × 10**^**−27**^	8.59 × 10^−1^	7.52 × 10^−2^	**1.65 × 10**^**−30**^

BP, blood pressure; DBP, diastolic blood pressure; EU, European; MAGMA, Multi-marker Analysis of GenoMic Annotation; SBP, systolic blood pressure.

This table shows the Beta, SE, and P-value for the MAGMA integration of GWAS summary statistics of EU-Meta-BP traits with human mature kidney single-cell RNA sequencing data set. Nominally significant associations (*P* < 0.05) are shown in bold.

## Discussion

A major challenge in genomics of complex traits, including BP, is the biological interpretation of thousands of identified genomic variant associations. Previous efforts to deal with this challenge were focused on prioritizing genes, mechanisms, and relevant tissues.^1^, ^2^, ^3^ However, prioritization of relevant cell types underlying the control of BP warrants further investigations. In this study, we attempted to connect human genomic findings for BP to specific kidney cell types defined by their scRNA-seq expression profiles. We prioritized kidney cell types with which the common genetic variants identified for BP showed the best “fit.”

We used human kidney scRNA-seq data for cell-type prioritization. Our analyses prioritized 2 specific cell types for BP traits, namely myofibroblasts and endothelial cells, from mature human kidneys. More specifically, SBP heritability was enriched in myofibroblast cells from the stroma compartment and DBP heritability was enriched in descending vasa recta endothelial cells, peritubular capillary endothelial cells and myofibroblast cells from the stroma compartment, all from the kidney cortex. The association of endothelial cells of the distal tubule segment from the mouse kidney cortex with SBP was previously reported.^9^ Endothelial cells were also nominally significant in our study for SBP and Bonferroni significant for DBP. However, the highly significant association of myofibroblast cells with both SBP and DBP from the stroma compartment is novel. In addition, our discovery is based on the human kidney, encompassing a great variety of cell types, and the ever-largest GWAS of BP to date, compared to mouse kidney and smaller GWASs used in previous studies. This may imply why some previous findings were not similarly prioritized.

We then replicated this novel finding using different independent data sets as well as by applying an alternative computational method. Among these replication efforts, the novel association of myofibroblast cells was replicated in the fetal human kidney at 7 to 16 weeks postconception. In a study of fetal kidney development stages from 10 to 30 weeks postconception, Almeida *et al.* reported that the development of the distal and proximal tubules as well as the glomeruli occurs in the cortex at week 10 postconception and other kidney sections of the fetus; that is, medulla and pelvic, develop afterward.^21^ Doppler ultrasound investigations of cardiovascular physiology in lamb fetuses of 13 to 41 weeks established fetal cardiac output and distribution of blood flow in fetuses of 13 weeks.^22^ Assuming a similar development sequence in humans, the successful replication of our findings in the fetal data set suggests that the myofibroblast contribution to BP regulation starts with cardiovascular system development.

Myofibroblast cells are the activated type of fibroblast cells and express profibrotic markers such as collagen type I which shows a positive correlation with BP.^23^^,^^24^ These cells express a regulator of G-protein signaling, RGS5, loss of which results in hypertension and hyperresponsiveness to vasoconstrictors and vascular stiffening, which indicates a causal role for RGS5 in vascular homeostasis.^25^

An scRNA-seq study of proximal tubule and nonproximal tubule cells from healthy and fibrotic human kidneys revealed distinct subpopulations of fibroblasts and pericyte cells as the origins of kidney myofibroblasts.^26^ Interestingly, a class of mesenchymal stromal cells in the kidney, known as mesangial cells, are reported to be distinct from fibroblasts and demonstrate a close similarity with myofibroblasts by upregulating both *ACTA2* (a special marker for myofibroblasts) and *RGS5*. In the adult kidney, mesangial cells support glomerular capillaries, and outside of the glomerulus as recently identified in an area known as the extraglomerular mesangium. Extraglomerular mesangial cells are involved in regulating BP and fluid volume through secretion of renin. In contrast, mesangiolysis, characterized by injury and loss of mesangial cells, has been observed in ischemia and hypertension.^23^

Angiotensin 2–induced hypertension,^27^ may promote the phenotypic change of fibroblasts to myofibroblasts that may proliferate and invade the periglomerular and peritubular spaces, which contribute to matrix deposition with excessive generation of extracellular matrix proteins such as collagens in the tubulointerstitial area.^28^^,^^29^

Attenuating angiotensin 2 generation might be effective to reduce myofibroblasts-induced fibrosis in hypertensive heart disease. In rats, pharmacological stimulation of soluble guanylate cyclase decreased myofibroblast transformation, collagen accumulation in the left ventricle, and gene expressions of transforming growth factor-β1 and type 1 collagen.^30^^,^^31^ These explanations may indicate fibroblast to myofibroblast transition as a key mechanism in hypertension.

Nevertheless, by leveraging Mendelian randomization methodology, our study adds a primary causal role for myofibroblasts in BP regulation, to its previously known involvement in injury response to high BP. The findings suggest that during normal heartbeats, myofibroblasts play a role in causing a slight increase in arterial pressure, especially during SBP, which helps maintain blood distribution balance, particularly in the kidneys. It is conceivable that as blood flows to the kidneys, pressure naturally decreases, and myofibroblasts act as a negative feedback mechanism, amplifying systolic pressure. However, further investigation is needed to understand the underlying mechanism. As hypertension progresses, myofibroblasts intensify this process, leading to organ fibrosis, notably in the kidneys. This highlights a positive feedback mechanism in the progression of organ fibrosis. The presence of both positive and negative feedback loops within the BP regulation mechanism, involving myofibroblasts, suggests that this cell type is a highly promising candidate for managing hypertension. Myofibroblast involvement in the regulation of BP has also been observed in organs other than the kidney such as heart and adipose tissues. In a previous study, Jagadeesh *et al.*^32^ have reported associations of pericyte and smooth muscle cell types from heart tissue (*n* = 12 cell types) with SBP and DBP based on UK Biobank GWASs (*n* = ∼420,000) by utilizing the sc-linker method. Although their scRNA-seq data set was lacking myofibroblasts, it is noteworthy that vascular pericytes or smooth muscle cells are among precursor cells of myofibroblasts.^33^ Because BP traits are heritable, and variants influencing these traits in the genome are disseminated throughout all body tissues following meiotic divisions during gametogenesis and subsequent mitotic divisions, many body tissues can be involved in the regulation of the phenotype associated with these variants.^34^

However, as mentioned, our primary focus is on the kidney, a key organ for the regulation of BP, utilizing the largest BP GWAS to date (n > 1 million). Further analyses prioritizing cell types in other cardiovascular tissues are imperative to complete the regulatory map of BP.

The importance of perivascular adipose tissue, which is an adipose tissue depot surrounding human blood vessels, has also been previously shown because it maintains and regulates the structure and function of the normal arterial wall through both autocrine and paracrine mechanisms.^35^ However, little is known about the contribution of myofibroblasts in adipose tissue. Ruan and colleagues investigated the role of perivascular adipose tissue in the regulation of adventitial fibroblast function and showed that in DOCA-salt hypertensive rat models compared to controls, perivascular collagen density and adventitial *ACTA2* expression increased, suggesting that perivascular adipose tissue progenitors differentiate into myofibroblasts.^36^ Although there is evidence regarding the association of adipose tissue myofibroblasts with BP in mice, we acknowledge that there is no evidence regarding the impact of adipose tissue myofibroblasts on BP in humans.

In addition, it has been observed in heart interstitial fibrosis caused by hypertension that myofibroblasts upregulated *ACTA2* expression, secrete large amounts of matrix proteins, and form a collagen-based scar.^37^^,^^38^ These as well as yet to be discovered mechanisms may explain the replication of myofibroblast cell type from other organs, in scRNA-seq data sets lacking kidney myofibroblasts.

Despite our meticulous use of 2 different statistical methods, multiple independent scRNA-seq data sets, and use of BP GWAS results of another ethnicity to avoid any spurious findings, the following considerations must be taken into account. First, although further analyses on the fetal and the mature human kidney data sets confirmed the original finding of kidney myofibroblasts, these cells were absent in the Tabula Sapiens^11^ and Tabula Muris^12^ scRNA-seq data sets. This is likely due to the wide variety of organs they include, and the smaller number of cells and fewer clusters detected there in each specific organ. However, myofibroblasts of other organs such as fat and heart were still replicated using these 2 data sets. Second, the overall assumption behind the ES calculation and therefore cell-type prioritization based on this metric is that the set of trait-causing genes must increase expression in the given cell type.^14^ Although this assumption is widely used, cell-type specificity might not only be driven by higher gene expression.^34^ Third, this approach assumes that specific patterns of gene expression in cell types are highly homologous between humans and mice.^14^ Several studies have already investigated and demonstrated this similarity while conserving a place for potential divergence between mice and humans.^39^^,^^40^ Altogether, additional work warrants to further confirm the prioritized cell types relevant to hypertension. Moreover, further exploration of individual loci could provide valuable insights into potential gene candidates in myofibroblasts. In addition, integrating scRNA-Seq/spatial gene expression, epigenomic, and GWAS data sets helps to link the inferred representation of the underlying biological processes beyond cell types.

In sum, our results, followed and validated by multiple replication efforts, provide novel evidence that myofibroblast cells play a key role in regulation of BP, offering promise for future follow-up studies shedding light on its contribution in regulating BP.

## Appendix

### List of International Consortium of Blood Pressure

Evangelos Evangelou, Helen R. Warren, He Gao, Georgios Ntritsos, Niki Dimou, Tonu Esko, Reedik Mägi, Lili Milani, Peter Almgren, Thibaud Boutin, Stéphanie Debette, Jun Ding, Franco Giulianini, Elizabeth G. Holliday, Anne U. Jackson, Ruifang Li -Gao, Wei -Yu Lin, Jian'an Luan , Massimo Mangino, Christopher Oldmeadow, Bram Peter Prins, Yong Qian, Muralidharan Sargurupremraj, Nabi Shah, Praveen Surendran, Sébastien Thériault, Niek Verweij, Sara M. Willems, Jing -Hua Zhao, Philippe Amouyel, John Connell, Renée de Mutsert, Alex S. F. Doney , Martin Farrall, Cristina Menni, Andrew D. Morris, Raymond Noordam, Guillaume Paré, Neil R. Poulter, Denis C. Shields, Alice Stanton, Simon Thom, Gonçalo Abecasis, Najaf Amin, Dan E. Arking, Kristin L. Ayers, Caterina M. Barbieri, Chiara Batini, Joshua C. Bis, Tineka Blake, Murielle Bochud, Michael Boehnke, Eric Boerwinkle, Dorret I. Boomsma, Erwin P. Bottinger, Peter S. Braund, Marco Brumat, Archie Campbell, Harry Campbell, Aravinda Chakravarti, John C. Chambers, Ganesh Chauhan, Marina Ciullo, Massimiliano Cocca, Francis Collins, Heather J. Cordell, Gail Davies, Martin H. de Borst, Eco J. de Geus, Ian J. Deary, Joris Deelen, Fabiola Del Greco M., Cumhur Yusuf Demirkale, Marcus Dörr, Georg B. Ehret, Roberto Elosua, Stefan Enroth, A. Mesut Erzurumluoglu, Teresa Ferreira, Mattias Frånberg, Oscar H. Franco, Ilaria Gandin, Paolo Gasparini, Vilmantas Giedraitis, Christian Gieger, Giorgia Girotto, Anuj Goel, Alan J. Gow, Vilmundur Gudnason, Xiuqing Guo, Ulf Gyllensten, Anders Hamsten, Tamara B. Harris, Sarah E. Harris, Catharina A. Hartman, Aki S. Havulinna, Andrew A. Hicks, Edith Hofer, Albert Hofman, Jouke-Jan Hottenga, Jennifer E. Huffman, Shih-Jen Hwang, Erik Ingelsson, Alan James, Rick Jansen, Marjo -Riitta Jarvelin, Roby Joehanes, Åsa Johansson, Andrew D. Johnson, Peter K. Joshi, Pekka Jousilahti, J Wouter Jukema, Antti Jula, Mika Kähönen, Sekar Kathiresan, Bernard D. Keavney, Kay-Tee Khaw, Paul Knekt, Joanne Knight, Ivana Kolcic, Jaspal S. Kooner, Seppo Koskinen, Kati Kristiansson, Zoltan Kutalik, Maris Laan, Marty Larson, Lenore J. Launer, Benjamin Lehne, Terho Lehtimäki, David C. M. Liewald, Li Lin, Lars Lind, Cecilia M. Lindgren, YongMei Liu, Ruth J. F. Loos, Lorna M. Lopez, Yingchang Lu, Leo-Pekka Lyytikäinen, Anubha Mahajan, Chrysovalanto Mamasoula, Jaume Marrugat, Jonathan Marten, Yuri Milaneschi, Anna Morgan, Andrew P. Morris, Alanna C. Morrison, Peter J. Munson, Mike A. Nalls, Priyanka Nandakumar, Christopher P. Nelson, Teemu Niiranen, Ilja M. Nolte, Teresa Nutile, Albertine J. Oldehinkel, Ben A. Oostra, Paul F. O'Reilly, Elin Org, Sandosh Padmanabhan, Walter Palmas, Aarno Palotie, Alison Pattie, Brenda W. J. H. Penninx, Markus Perola, Annette Peters, Ozren Polasek, Peter P. Pramstaller, Quang Tri Nguyen, Olli T. Raitakari, Rainer Rettig, Kenneth Rice, Paul M. Ridker, Janina S. Ried, Harriëtte Riese, Samuli Ripatti, Antonietta Robino, Lynda M. Rose, Jerome I. Rotter, Igor Rudan, Daniela Ruggiero, Yasaman Saba, Cinzia F. Sala, Veikko Salomaa, Nilesh J. Samani, Antti-Pekka Sarin, Reinhold Schmidt, Helena Schmidt, Nick Shrine, David Siscovick, Albert V. Smith, Harold Snieder, Siim Sõber, Rossella Sorice, John M. Starr, David J. Stott, David P. Strachan, Rona J. Strawbridge, Johan Sundström, Morris A. Swertz, Kent D. Taylor, Alexander Teumer, Martin D. Tobin, Maciej Tomaszewski, Daniela Toniolo, Michela Traglia, Stella Trompet, Jaakko Tuomilehto, Christophe Tzourio, André G. Uitterlinden, Ahmad Vaez, Peter J. van der Most, Cornelia M. van Duijn, Germaine C. Verwoert, Veronique Vitart, Uwe Völker, Peter Vollenweider, Dragana Vuckovic, Hugh Watkins, Sarah H. Wild, Gonneke Willemsen, James F. Wilson, Alan F. Wright, Jie Yao, Tatijana Zemunik, Weihua Zhang, John R. Attia, Adam S. Butterworth, Daniel I. Chasman, David Conen, Francesco Cucca, John Danesh, Caroline Hayward, Joanna M. M. Howson, Markku Laakso, Edward G. Lakatta, Claudia Langenberg, Olle Melander, Dennis O. Mook-Kanamori, Colin N. A. Palmer, Lorenz Risch, Robert A. Scott, Rodney J. Scott, Peter Sever, Tim D. Spector, Pim van der Harst, Nicholas J. Wareham, Eleftheria Zeggini, Daniel Levy, Patricia B. Munroe, Christopher Newton-Cheh, Morris J. Brown, Andres Metspalu, Bruce M. Psaty, Louise V. Wain, Paul Elliott and Mark J. Caulfield

## Disclosure

All the authors declared no competing interests.

## Supplementary Material

Supplementary File (PDF)

**Table S1.** Full results of discovery analysis listed as prioritized cell types estimated by LDSC in blood pressure related phenotypes.
